# Genomic profiling and clinical utility of circulating tumor DNA in metastatic prostate cancer: SCRUM-Japan MONSTAR SCREEN project

**DOI:** 10.1038/s44276-024-00049-7

**Published:** 2024-04-03

**Authors:** Masaki Shiota, Nobuaki Matsubara, Taigo Kato, Masatoshi Eto, Takahiro Osawa, Takashige Abe, Nobuo Shinohara, Koshiro Nishimoto, Yota Yasumizu, Nobuyuki Tanaka, Mototsugu Oya, Takao Fujisawa, Satoshi Horasawa, Yoshiaki Nakamura, Takayuki Yoshino, Norio Nonomura

**Affiliations:** 1 Department of Urology, Graduate School of Medical Sciences, Kyushu University, Kyushu, Japan; 2 Department Medical Oncology, National Cancer Center Hospital East, Chiba, Japan; 3 Department of Urology, Osaka University Graduate School of Medicine, Yamadaoka, Japan; 4 Department of Urology, Graduate School of Medicine Hokkaido University, Hokkaido, Japan; 5 Department of Uro-Oncology, Saitama Medical University International Medical Center, Saitama, Japan; 6 Department of Urology, Keio University School of Medicine, Tokyo, Japan; 7 Department Head and Neck Medical Oncology, National Cancer Center Hospital East, Chiba, Japan; 8 Translational Research Support Office, National Cancer Center Hospital East, Chiba, Japan; 9 Department of Gastroenterology and Gastrointestinal Oncology, National Cancer Center Hospital East, Chiba, Japan

## Abstract

**Background:**

Circulating tumor DNA (ctDNA) testing has emerged as a novel tool for cancer precision medicine. This study investigated the genomic profiling and clinical utility of ctDNA in metastatic prostate cancer.

**Methods:**

This is a nation-wide prospective observational study. Patients treated with systemic treatment for metastatic castration-sensitive prostate cancer (mCSPC) and metastatic castration-resistant prostate cancer (mCRPC) were included. ctDNA was analyzed using FoundationOne Liquid®CDx at enrollment. In a subset of patients, ctDNA after disease progression and tissue prior to the initiation of treatment were examined using FoundationOne Liquid®CDx and FoundationOne®CDx, respectively.

**Results:**

The frequency of *AR* alterations and homologous recombination repair (HRR) defect was higher in mCRPC compared with mCSPC. Tumor mutational burden was correlated between tissue and ctDNA at pre-treatment, as well as ctDNA between at pre-treatment and at post-treatment. Patients with HRR defect were associated with shorter time to castration resistance in androgen deprivation therapy/combined androgen blockade, but not in androgen receptor pathway inhibitor, compared with patients without HRR defect in mCSPC. Time to treatment failure in patients with *AR* amplification or *AR* mutation was shorter compared with patients without *AR* alterations in mCRPC.

**Conclusions:**

This study revealed valuable findings for the clinical care of metastatic prostate cancer. Especially, predictive factors such as HRR defect in mCSPC should be validated in the future.

## Introduction

Prostate cancer is one of the most diagnosed cancers among men in Western countries [1]. Metastatic castration-sensitive prostate cancer (mCSPC) can occur as *de novo* disease or due to recurrence after radical local treatment for localized prostate cancer, representing approximately 10% of newly diagnosed cases in Japan [2]. Although 1st line treatment including androgen deprivation therapy (ADT) is initially highly effective in the relief of cancer-related symptoms, prostate-specific antigen (PSA) decline, and tumor shrinking, therapeutic resistance is almost universal, and most mCSPC progresses to castration-resistant prostate cancer (CRPC). In recent years, many drugs for treating patients with mCSPC and CRPC have been developed in landmark trials and have become available [3, 4]. Recently, several novel treatments including poly-ADP ribose polymerase inhibitors and immune checkpoint inhibitors based on genomic findings have been developed for the treatment of metastatic CRPC (mCRPC), realizing cancer precision medicine [5–7]. However, most treatment decisions are currently not performed on the basis of individual genomic profiling in any state of progressive disease, except PARP inhibitors for mCRPC.

Circulating tumor DNA (ctDNA) testing has emerged as a novel tool to drive cancer precision medicine. Although next-generation sequencing (NGS) of tumor tissue is the optimal method for comprehensive genomic profiling, accessing metastatic sites for tissue biopsy is challenging. In addition, the tumor tissue from only one region may not be sufficient to capture intra-patient tumor heterogeneity. In contrast, ctDNA can be obtained less invasively and repeatedly, and capture the current genomic profile of the tumor, encompassing its heterogeneity [8–10]. So far, evidence of ctDNA analysis has been accumulating in clinical settings, which revealed the genomic landscape and showed concordance with genomic profiles detected in tissues as well as prognostic value in advanced prostate cancer [11–13]. In addition, clonal evolution by treatment pressure was reported among patients mainly with mCRPC [14–19]. In contrast, their roles in mCSPC are relatively limited, and no study has reported clonal evolution in paired ctDNA of mCSPC [17, 20–22]. Furthermore, the predictive value of genomic alterations on treatment outcomes has rarely been reported, which is useful in making decisions on treatment choice in the clinical setting.

Therefore, we conducted a prospective observational study that investigated genomic profiling and the clinical utility of ctDNA in metastatic prostate cancer.

## Materials and methods

### Patient enrollment

SCRUM-Japan MONSTAR SCREEN is a nationwide study involving core cancer institutions in Japan investigating ctDNA genomic profiling and gut microbiome; Six institutions were involved in the MONSTAR-Urology subgroup (National Cancer Center Hospital East, Osaka University Hospital, Kyushu University Hospital, Hokkaido University Hospital, Keio University Hospital, and Saitama Medical University International Medical Center).

The key inclusion criteria were as follows: (1) histopathologically confirmed unresectable or metastatic solid cancer, (2) receipt of or planned following systemic therapy; (cohort A) 1st line treatment, (cohort B) treatment after pre-defined genomic alterations were identified, (cohort C) immune checkpoint inhibitors, and (cohort D) pre-defined androgen receptor pathway inhibitors (ARPI) including abiraterone and enzalutamide, (3) patient aged ≥ 16 years, (4) Eastern Cooperative Oncology Group performance status of 0–1; (5) adequate organ function; and (6) receipt of or planned cancer genomic profiling test using tumor tissue. Among them, patients who were diagnosed with metastatic prostate cancer and enrolled from the institutions of the MONSTAR-Urology subgroup were the subjects in this study. Patients whose ctDNA at pre-treatment undetectable were excluded.

This study was conducted in accordance with the Declaration of Helsinki and the Japanese Ethical Guidelines for Medical and Health Research Involving Human Subjects. Eligible patients provided written informed consent. The study protocol was approved by the Institutional Review Board of each participating institution and registered at the University Hospital Medical Information Network (UMIN) Clinical Trials Registry (protocol nos. UMIN000036749). This study was initiated in August 2019 and enrollment was completed in February 2022.

### ctDNA genotyping

Blood sampling were performed before corresponding treatment including ADT in mCSPC (pre-treatment) and after progression (post-treatment). NGS analysis of ctDNA was performed using FoundationOne Liquid®CDx (F1LCDx®) at a Clinical Laboratory Improvement Amendments (CLIA)-certified, College of American Pathologists (CAP)-accredited laboratory designated by Foundation Medicine Inc., as described [23, 24]. The assay uses hybrid-capture technology and deep sequencing coverage to report single nucleotide variants, indels, genomic rearrangements, copy number variations (CNVs) (amplifications and losses) in 324 cancer-related genes, and genomic signatures including blood tumor mutational burden (bTMB), microsatellite instability (MSI), and tumor fraction (TF) [25]. bTMB-High was defined by ≥14 mut/Mb, and TF was considered elevated when it could be estimated using aneuploidy, a threshold of ≥ 10% [25, 26].

Homologous recombination repair (HRR) defect was defined by alterations in 15 HRR genes (*BRCA1*, *BRCA2*, *ATM*, *BARD1*, *BRIP1*, *CDK12*, *CHEK1*, *CHEK2*, *FANCL*, *PALB2*, *PPP2R2A*, *RAD51B*, *RAD51C*, *RAD51D*, and/or *RAD54L*). Mismatch repair (MMR) deficient was defined by alterations in 4 MMR genes (*MLH1*, *MSH2*, *MSH6*, and/or *PMS2*). Oncogenic alterations were grouped by major signaling pathways from pan-cancer analyses, excluding the HIPPO, NRF2 and TGFβ pathways that were altered in < 1% of samples within clinical subgroups [21, 27]. TP53 was not grouped with DNA repair genes and considered separately, as were two individual genes relevant in prostate cancer (AR, SPOP) (Supplementary Table 1).

### Tumor tissue genotyping

NGS analysis of tumor tissue was performed using FoundationOne®CDx (F1CDx®) at a CLIA-certified, CAP-accredited laboratory designated by Foundation Medicine Inc., as described [23, 28]. The pathologic diagnosis of tissue biopsy was confirmed on routine hematoxylin and eosin–stained slides, and all samples forwarded for DNA extraction contained a minimum of 20% tumor nuclei. 50 − 1000 ng of DNA are used for whole genome shotgun library construction and hybrid-capture. F1CDx® covers 324 cancer-related genes and detects relevant single nucleotide variants, CNVs, gene fusions and indels, and genomic signatures including tumor mutational burden (TMB) and MSI. Assessment of TMB and MSI was performed as described previously [25].

### Clinical data

The clinicopathological information and efficacy data relating to the systemic therapy of patients were collected prospectively using an electronic data capture system. These clinical data and genotyping results were stored in a clinical-grade database and used for an integrated clinico-genomic analysis.

All patients were treated under physician’s discretion according to current guideline. ADT was performed by surgical and medical castration with gonadotropin releasing hormone agonist (leuprorelin and goserelin) or antagonist (degarelix). Combined androgen blockade (CAB) was defined as combination therapy with ADT plus first-generation antiandrogen including bicalutamide and flutamide. In August 2019 when this study started, abiraterone for high-risk mCSPC defined by Latitude criteria and mCRPC, as well as enzalutamide and docetaxel for mCRPC had been approved in Japan. However, enzalutamide and apalutamide were approved in May 2020 for mCSPC in Japan. Progression to CRPC was defined according to Prostate Cancer Working Group 2 (PCWG2) criteria [29]. For the analysis of time to CRPC, time to treatment failure (TTF) and overall survival (OS), progression to castration resistance, treatment discontinuation, and death from any cause were defined as end events, respectively. Patients who did not experience any of these events were censored at the last follow-up visit. For the analysis of survival, the number of months from enrollment date to the earliest event or censoring date was calculated.

### Statistical analyses

All statistical analyses were performed using JMP16 software (SAS Institute, Cary, NC, USA). Continuous and categorical data were presented as median with interquartile range (IQR) and number with percentage, respectively. Comparison between groups of continuous data of matched samples and categorical data were analyzed using paired *t*-test and Fisher’s exact test, respectively. Correlations between parameters were determined using the Pearson’s correlation coefficients. Survival analysis was performed using the Kaplan–Meier method and log-rank test. Univariate and multivariate analyses were performed using the Cox hazard proportional model to estimate hazard ratios (HRs) with 95% confidence intervals (CIs), respectively. Differences on the prognostic impact between treatments were investigated through interaction tests. All *P*-values were two-sided. *P*-values < 0.05 were considered significant.

## Results

### Genomic landscape in ctDNA at pre-treatment

**Fig. 1:**
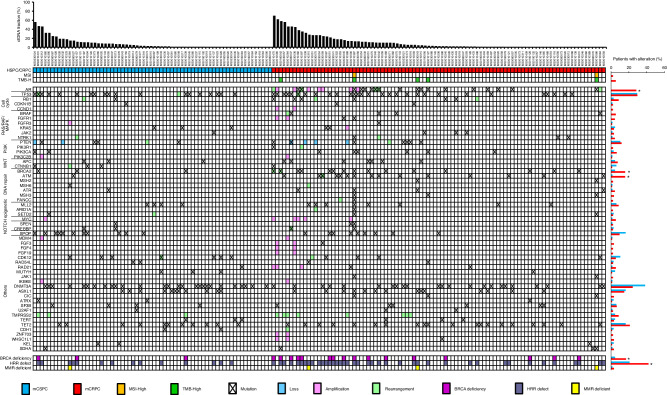
Genomic landscape by F1LCDx® in metastatic prostate cancer. Genomic alterations observed in multiple cases with variant allele frequency of over 2% were listed. Patients are sorted by tumor fraction (top). Genes are grouped by pathway. Frequency of alterations in each gene among 68 patients with metastatic castration-sensitive prostate cancer (mCSPC) and 95 patients with metastatic castration-resistant prostate cancer (mCRPC) is provided on the right. *statistical significance (mCSPC vs mCRPC).

In total, 192 patients were enrolled from six institutions to participate in MONSTAR-Urology subgroup, and 163 patients were analyzed after excluding either unavailable clinical data (*n* = 3), non-metastatic disease (*n* = 13), or ctDNA undetectable (*n* = 12), and both non-metastatic and ctDNA undetectable (*n* = 1) (Supplementary Fig. 1). Among them, 68 patients were mCSPC and 95 patients were mCRPC. Genomic alterations and landscape of ctDNA by F1LCDx® obtained at enrollment to MONSTAR SCREEN were demonstrated according to TF by mCSPC and mCRPC (Fig. 1, Supplementary Table 2). MSI-High were reported in 2 cases (2.1%) with mCRPC, who accompanied *MSH2* or *MSH3* mutation and both were bTMB-High. bTMB-High were not observed in cases with mCSPC, but detected in 5 (5.3%) cases with mCRPC, who carried HHR defect and/or MMR deficient. Among various pathogenic and likely pathogenic alterations, genomic alterations observed in multiple cases with variant allele frequency (VAF) of over 2% were listed. *TP53* alteration that was most frequently observed was detected in 29.4% and 29.5% with mCSPC and mCRPC, respectively. Also, *PTEN* alteration was common abnormality, which was observed in 10.3% and 11.6% of mCSPC and mCRPC, respectively. The alteration frequency in *AR* gene including single nucleotide variant, amplification, and genomic rearrangement was higher in mCRPC (28.4%) compared with mCSPC (0%, *P* < 0.0001). *BRCA1*, *BRCA2*, and *ATM* alterations were detected in 3 [1 case (1.5%) with mCSPC, 2 cases (2.1%) with mCRPC], 18 [3 case (4.4%) with mCSPC, 15 cases (15.8%) with mCRPC], and 16 [1 case (1.5%) with mCSPC, 15 cases (15.8%) with mCRPC] cases, respectively. *BRCA2* and *ATM* alterations were more frequent in mCRPC compared with mCSPC (*BRCA2*, *P* = 0.024; *ATM*, *P* = 0.0023). Similarly, HRR defect were observed in 20.6 and 42.1% of mCSPC and mCRPC (*P* = 0.0043).

### Concordance between tissue and ctDNA

**Fig. 2:**
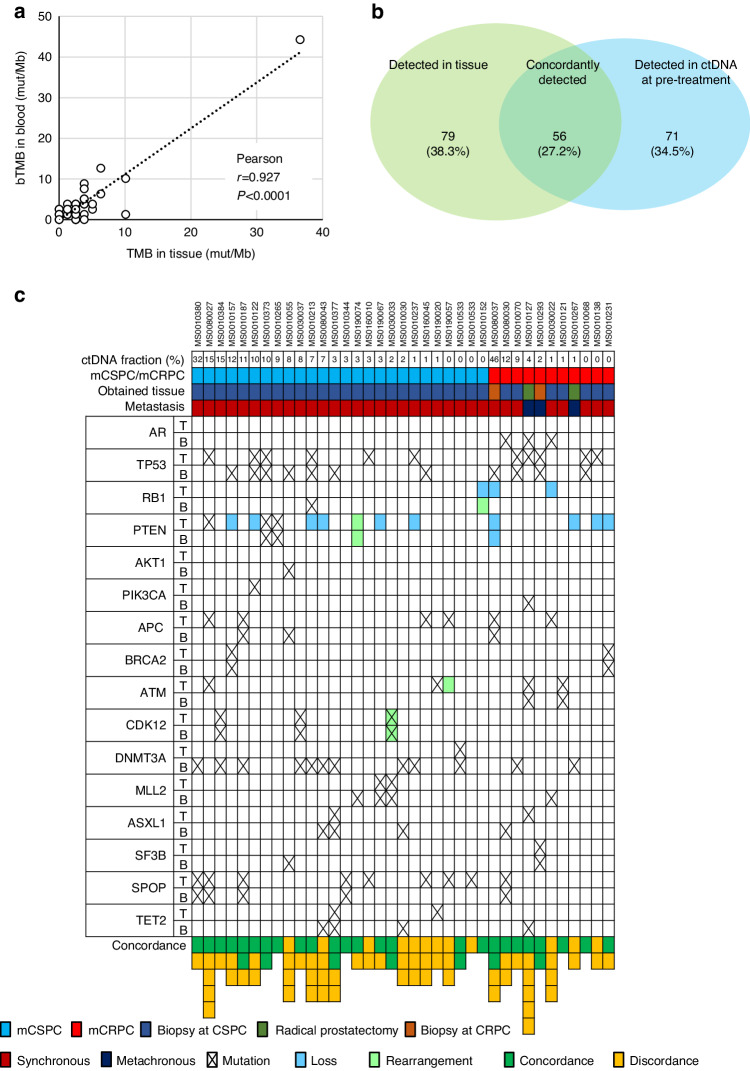
Concordance between tissue and ctDNA. **a** Correlation of tumor mutational burden (TMB) in tissue and blood TMB (bTMB) in ctDNA from matched patients (*n* = 37). **b** Venn diagram on of concordance and discordance of genomic alterations between paired tissue and ctDNA from matched patients (*n* = 38). **c** Altered genes with frequency of over 5% in paired tissue or ctDNA from matched patients (*n* = 38). T tumor, B blood.

To investigate the concordance between tissue and ctDNA at pre-treatment, 49 archival and 2 fresh tissues were analyzed by F1CDx®. However, F1CDx® testing failed in 13 tissues, resulting in concordance having been examined in 38 patients (Supplementary Fig. 1). TMB in tissue and bTMB in ctDNA showed high correlation (*r* = 0.927, *P* < 0.0001, Fig. 2a). When concordance of gene alterations was examined, 56 (27.2%) of gene alterations were concordantly observed between tissue and ctDNA. However, 79 (38.3%) and 71 (34.5%) of gene alterations were detected only in tissue and ctDNA, respectively. The sensitivity of ctDNA testing represented by the ratio of detected gene alterations in blood among those in tissues was 41.5% (44.8% among 19 samples with TF ≥ 1%) (Fig. 2b). The sensitivities of ctDNA testing in mCSPC and mCRPC were 43.8% and 35.9%, respectively. The sensitivities of ctDNA testing of mutation, CNV and rearrangement were 51.1%, 15.4% and 62.5%, respectively. Figure 2c demonstrated the concordance status of alterations in the genes with a frequency of over 5% in tissue or ctDNA at pre-treatment among matched patients. Among them, 32 (33.7%) alterations were concordant between tissue and ctDNA, whereas 63 (66.3%) alterations were discordant. *AR* alterations were detected only in ctDNA from patients with mCRPC, whereas there was no *AR* alteration in tissue, which was mostly obtained before hormonal therapy. Meanwhile, copy number loss of *RB1* and *PTEN* were detected in tissue, but not detected in blood samples with ctDNA fraction of less than 15%. Similarly, *APC*, *ATM*, and *SPOP* alterations in tissue were not detected in ctDNA in some patients. Meanwhile, *TP53*, *DNMT3A*, *ASXL1*, and *TET2* alterations in ctDNA were not detected in tissue in some patients, presumably because of clonal hematopoiesis of indeterminate potential. Alterations in *BRCA2* and *CDK12* were detected in both tissue and ctDNA in 2 and 3 patients, respectively.

### Clonal evolution during treatment

**Fig. 3:**
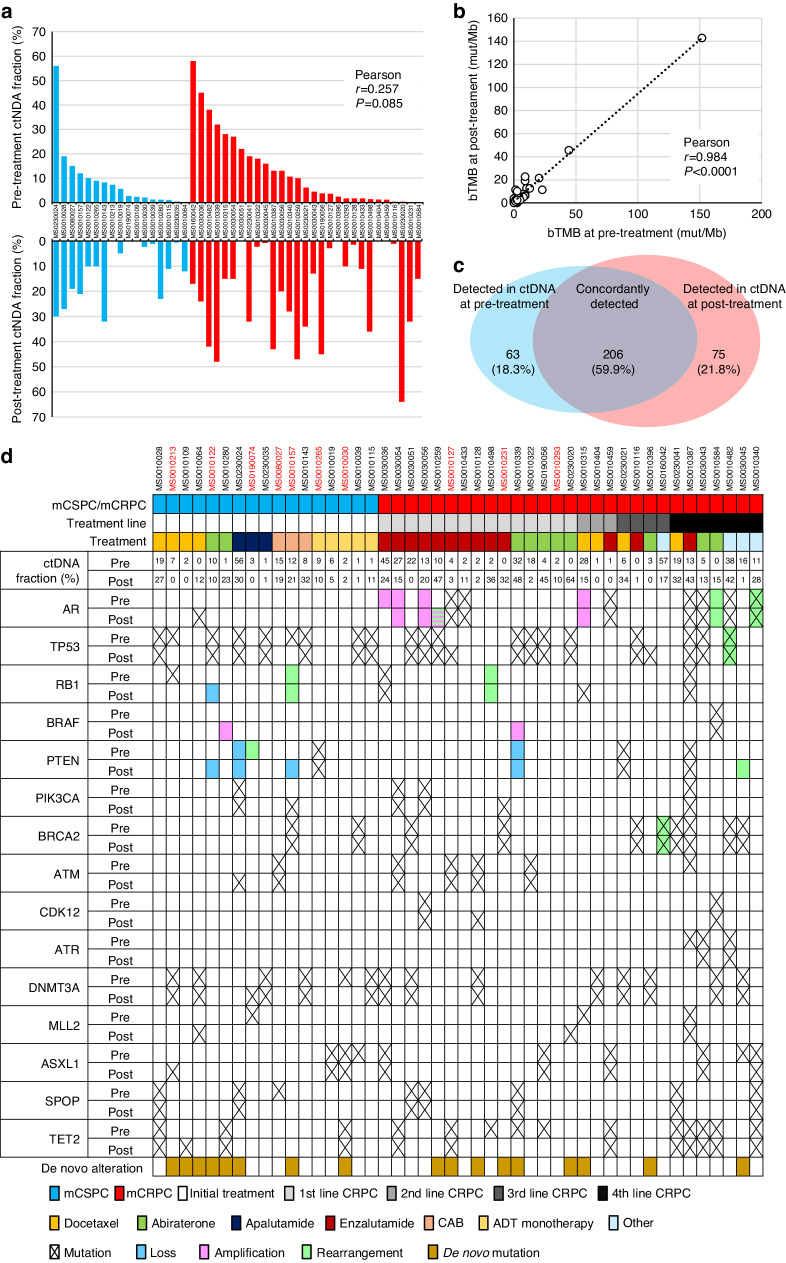
Clonal evolution during treatment. **a** Change of tumor fraction in paired ctDNA at pre-treatment and post-treatment from same patients (*n* = 46). Blue bar, metastatic castration-sensitive prostate cancer; red bar, metastatic castration-resistant prostate cancer. **b** Correlation of blood tumor mutation burden (bTMB) in paired ctDNA at pre-treatment and post-treatment from matched patients (*n* = 46). **c** Venn diagram on of concordance and discordance of genomic alterations in ctDNA between pre-treatment and post-treatment from matched patients (*n* = 46). **d** Altered genes with frequency of over 5% in ctDNA at pre-treatment or post-treatment from matched patients (*n* = 46). The data on tumor tissue genotyping was available in patients in red.

Next, genomic alterations in ctDNA between pre-treatment and post-treatment were compared in 46 patients in whom paired F1LCDx® were available (Supplementary Fig. 1). TF at pre-treatment and post-treatment was shown in Fig. 3a, indicating decreased and increased TF after treatment, and the correlation between pre-treatment and post-treatment is weak (*r* = 0.257, *P* = 0.085, Fig. 3a). When the correlation of TF between pre-treatment and post-treatment was analyzed by diagnosis status and time to progression, TF between pre-treatment and post-treatment was correlated in mCSPC (*r* = 0.590, *P* = 0.013), but not mCRPC (*r* = 0.125, *P* = 0.52), shorter time to progression (<6 months, *r* = 0.340, *P* = 0.14), and longer time to progression (≥ 6 months, *r* = 0.151, *P* = 0.46). However, bTMBs between pre-treatment and post-treatment were highly correlated (*r* = 0.984, *P* < 0.0001, Fig. 3b). When concordance of gene alterations was examined, 206 (59.9%) gene alterations were concordantly observed in ctDNA between pre-treatment and post-treatment. In comparison, 63 (18.0%) and 75 (21.8%) gene alterations were detected only in ctDNA at pre-treatment and post-treatment, respectively (Fig. 3c). When samples with TF ≥ 1% at both pre-treatment and post-treatment were analyzed, 172 (65.4%) gene alterations were concordantly observed in ctDNA between pre-treatment and post-treatment. In comparison, 46 (17.5%) and 45 (17.1%) gene alterations were detected only in ctDNA at pre-treatment and post-treatment, respectively. Figure 3d demonstrated concordance status of alterations in the genes with frequency of over 5% in ctDNA at pre-treatment or post-treatment among matched patients. Among frequently-altered genes, *de novo* alterations that was emerged after treatment were detected in 18 (39.1%) patients. *De novo* alterations of *AR*, *TP53*, *RB1*, *BRAF*, *PTEN*, and *MLL2* were recurrently observed in 2 (4.3%), 2 (4.3%), 2 (4.3%), 2 (4.3%), 3 (6.5%), and 2 (4.3%) patients, whereas *de novo* alteration of other genes was rare. However, *TP53* mutation (*n* = 1) and *PTEN* loss (*n* = 2) were observed in tissue in cases whose sequencing data in tissue was available, indicating those alterations was not detected at pre-treatment. Interestingly, *de novo* alterations were detected in 4 (80.0%) among 5 patients treated with ADT plus ARPI (abiraterone, enzalutamide, or apalutamide) for mCSPC, whereas only 2 (25.0%) among 8 patients treated with ADT/CAB. In addition, *de novo* alterations were detected in 4 (30.8%) among 13 patients and 3 (37.5%) among 8 patients after enzalutamide and abiraterone treatment for mCRPC, respectively.

### Prognostic and predictive impacts of genomic alterations in mCSPC

Next, we investigated prognostic and predictive impacts of genomic alterations in mCSPC. The clinicopathological characteristics of patients with mCSPC were shown in Table 1. Among 68 patients, 32 (47.1%), 28 (41.2%), and 8 (11.8%) patients were treated with ADT/CAB, ADT plus ARPI, and ADT plus docetaxel, respectively. The association of alteration signatures, altered genes and altered pathway with a frequency of > 5% were analyzed with time to CRPC, TF ≥ 10% as well as *TP53* and *PTEN* alterations and NOTCH pathway alteration were associated with shorter time to CRPC regardless of treatment (Supplementary Fig. 2, Supplementary Table 3).

**Table 1:** Characteristics in patients with metastatic castration-sensitive prostate cancer.

Characteristics	*n* = 68
Median age, years (IQR)	73 (69–79)
Median PSA level, ng/ml (IQR)	254 (72.4–992)
NA	1
ISUP grade group, *n* (%)	
≤ III	5 (7.4%)
IV	23 (33.8%)
V	40 (58.8%)
Metastasis, *n* (%)	
Synchronous	66 (97.1%)
Metachronous	2 (2.9%)
Prior local treatment, *n* (%)	
Absence	61 (89.7%)
Curative radiation	7 (10.3%)
Lymph node metastasis, *n* (%)	
Absence	23 (33.8%)
Presence	45 (66.2%)
Bone metastasis, *n* (%)	
Absence	13 (19.1%)
1	5 (7.4%)
2 or 3	10 (14.7%)
≥ 4	40 (58.8%)
Lung metastasis, *n* (%)	
Absence	57 (83.8%)
Presence	11 (16.2%)
Liver metastasis, *n* (%)	
Absence	66 (97.1%)
Presence	2 (2.9%)
Treatment, *n* (%)	
ADT monotherapy	22 (32.4%)
CAB	10 (14.7%)
ADT plus docetaxel	8 (11.8%)
ADT plus abiraterone	15 (22.1%)
ADT plus enzalutamide	2 (2.9%)
ADT plus apalutamide	11 (16.2%)

*ADT* androgen deprivation therapy, *CAB* combined androgen blockade, *IQR* interquartile range, *ISUP* International Sciety of Urological Pathology, *NA* not available, *PSA* prostate-specific antigen

**Fig. 4:**
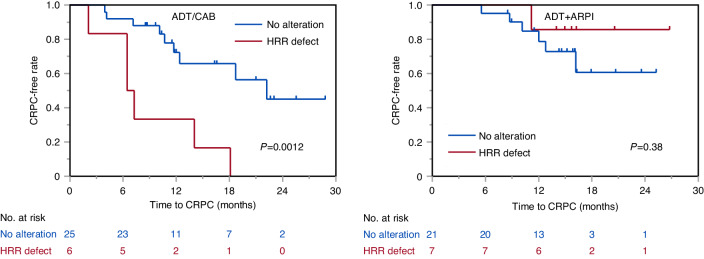
Predictive impacts of genomic alterations in metastatic castration-sensitive prostate cancer (mCSPC). Time to castration-resistant prostate cancer (CRPC) according to presence or absence of homologous recombination repair (HRR) defect in mCSPC patients treated with androgen deprivation therapy (ADT)/combined androgen blockade (CAB) (left) or ADT plus androgen receptor pathway inhibitor (ARPI) (right).

Subsequently, the predictive impact of genomic alterations in mCSPC between ADT/CAB and ADT plus ARPI was analyzed. Interestingly, the presence of HRR defect was differentially associated with time to CRPC between ADT/CAB and ADT plus ARPI (Supplementary Table 4). Patients with HRR defect were associated with shorter time to CRPC compared with patients without HRR defect when treated with ADT/CAB, whereas significant difference was not observed between patients with and without HRR defect when treated with ADT plus ARPI (Fig. 4). When adjusted by TF ( < 10% vs. ≥ 10%), metastatic burden; high burden defined by ≥ 4 bone metastasis or visceral metastasis (high vs low), and Gleason grade group (group ≤ 4 vs. 5), HRR defect was associated with shorter time to CRPC in ADT/CAB (HR, 6.12: 95% CI, 1.80–20.8; *P* = 0.0037), but not in ARPI (HR, 0.37: 95% CI, 0.043–3.20; *P* = 0.37). However, no pathway was predictive between ADT/CAB and ADT plus ARPI (data not shown).

### *AR* alterations in mCRPC

**Fig. 5:**
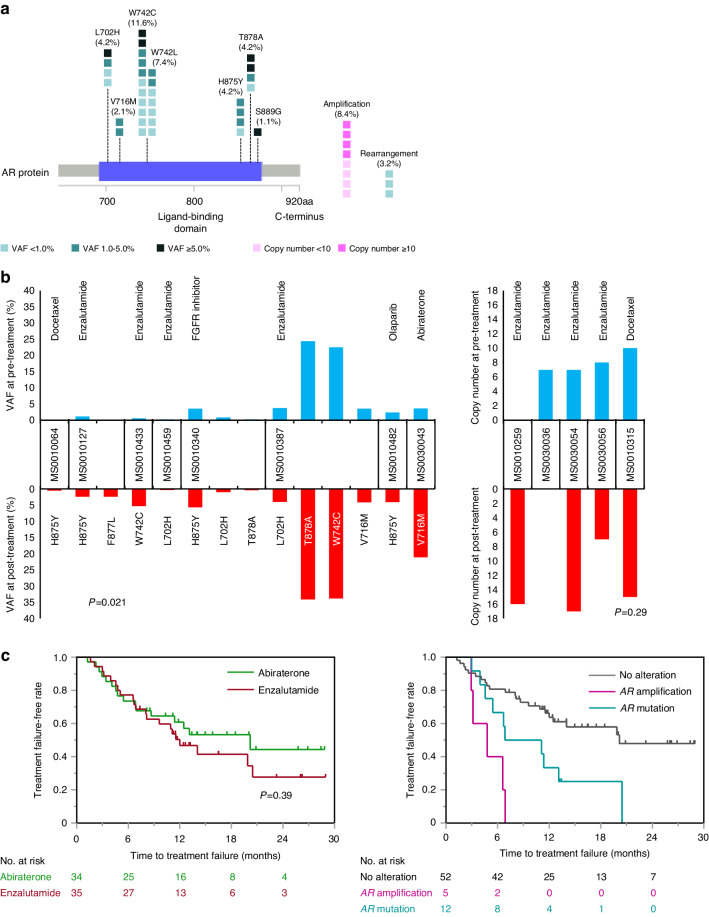
*AR* alterations in metastatic castration-resistant prostate cancer (mCRPC). **a** *AR* mutations, amplification, and rearrangement in F1LCDx® at pre-treatment in mCRPC (*n* = 95) are indicated. Each colored circle represents a single mutation with variant allele frequency (VAF) and copy number. **b** Change of VAF of *AR* mutation (left) and copy number of *AR* gene (right) in paired ctDNA at pre-treatment (blue bar) and post-treatment (red bar) from matched patients. **c** Time to treatment failure according to treatment (abiraterone or enzalutamide) (left), and *AR* gene alterations (right) in mCRPC patients.

Finally, we investigated the alteration status of *AR* gene among 95 patients with mCRPC (Table 2). At pre-treatment, *AR* W742C and W742L mutations were most frequently observed in 11 (11.6%) patients and 7 (7.4%) patients, followed by L702H (4.2%), H875Y (4.2%), and T878A (4.2%), who were treated without prior ARPI (*n* = 12) or with prior ARPI (*n* = 13) (Fig. 5a). In addition, *AR* gene amplification and rearrangement were detected in 8 (8.4%) and 3 (3.2%) patients. When *AR* alterations were compared in ctDNA between pre-treatment and post-treatment, VAF of *AR* mutation was increased after treatment (*P* = 0.021) although copy number of *AR* amplification was not increased statistically significantly (*P* = 0.29) (Fig. 5b). Interestingly, *AR* F877L mutation regarded as enzalutamide and/or apalutamide resistance emerged after enzalutamide treatment in one case (Fig. 5b). In addition, VAF of *AR* W742C mutation was increased after enzalutamide treatment in 2 cases (Fig. 5b). Next, the prognostic and predictive impacts of *AR* alterations were investigated among patients treated with ARPI for mCRPC within 3rd line treatment. Although TTF was comparable between abiraterone and enzalutamide, TTF in patients with *AR* amplification (*P* < 0.0001) or *AR* mutation (*P* = 0.012) was shorter compared with patients without *AR* alterations (Fig. 5c).

**Table 2:** Characteristics in patients with metastatic castration-resistant prostate cancer.

Characteristics	*n* = 95
Median age, years (IQR)	73 (68–76)
Median PSA level, ng/ml (IQR)	13.6 (3.62–70.0)
ISUP grade group, *n* (%)	
≤ III	13 (14.0%)
IV	25 (26.9%)
V	55 (59.1%)
NA	2
Lymph node metastasis, *n* (%)	
Absence	55 (57.9%)
Presence	40 (42.1%)
Bone metastasis, *n* (%)	
Absence	14 (14.7%)
1	18 (18.9%)
2 or 3	13 (13.7%)
≥ 4	50 (52.6%)
Lung metastasis, *n* (%)	
Absence	81 (85.3%)
Presence	14 (14.7%)
Liver metastasis, *n* (%)	
Absence	91 (95.8%)
Presence	4 (4.2%)
Treatment, *n* (%)	
Abiraterone	37 (38.9%)
Enzalutamide	38 (40.0%)
Taxane	11 (11.6%)
Others	9 (9.5%)
Treatment line for CRPC, *n* (%)	
1st line	61 (64.2%)
2nd line	13 (13.7%)
3rd line	7 (7.4%)
≥ 4th line	14 (14.7%)

*IQR* interquartile range, *ISUP* International Society of Urological Pathology, *NA* not available, *PSA* prostate-specific antigen

## Discussion

This study showed the genomic landscape of ctDNA, concordance between tissue and ctDNA, and clonal evolution during treatment in mCSPC and mCRPC. Consistently with previous studies, *TP53* was commonly detected, followed by alterations in *AR*, *PTEN* and HRR genes. Consistently with previous reports, the frequency of alterations in *AR* and HRR genes including *BRCA2* and *ATM* was enriched in mCRPC compared with mCSPC, suggesting that alterations in *AR* and HRR genes promote treatment resistance to initial ADT-based therapy in prostate cancer. Previously, Fan et al. have shown similar result that genomic alterations in *AR* and *CDK12* genes were enriched in ctDNA with mCRPC compared with those with *de novo* mCSPC among Asians [22].

This study showed modest concordance of 27.2% in all genes and 33.7% in frequently-altered genes between tissue and ctDNA. Previously, the concordance between tissue and ctDNA in matched patients was reported to be 75% in solid tumors including mainly lung cancer [30]. As well, Wyatt et al. reported high concordance between metastatic mCRPC tissue and blood synchronously sampled with tissue sampling in patients with TF of greater than 2% in ctDNA [12]. In this study, tissue sampling was performed from the primary region at treatment naïve in most cases. Therefore, several genomic alterations could be detected only in ctDNA, which may represent *de novo* alterations during spaciotemporal evolution. However, there is a possibility that clonal hematopoiesis of indeterminate potential was included in ctDNA samples because F1LCDx® does not include analysis of genomic DNA from normal cells. Inversely, genomic alterations detected only in tissues may represent current technical limitations in ctDNA analysis, as Chi et al. reported high concordance of *BRCA1*, *BRCA2*, and *ATM* alterations including nonsense, splice, and frameshift between tissue and ctDNA, but lower concordance for large rearrangement, and homozygous deletions [31]. In addition, *AR* gene alterations were detected only in ctDNA among the patients with mCRPC, which were not detectable in tissues from the primary site at treatment-naïve, representing one of the treatment resistant mechanisms to CRPC. These factors may have resulted in low concordance between tissue and ctDNA in this study.

This study evaluated clonal evolution during treatment using serial sampling in patients to determine therapy selection pressure. To the best of our knowledge, this is the first report on clonal evolution by paired ctDNA in mCSPC. Previously, Annala et al. reported clonal evolution in paired ctDNA before and after ARPI for mCRPC in a prospective study, indicating that most driver alterations were consistently detected, but about half of patients shifted in somatic populations after treatment [18]. Consistently, this study showed high concordance of genomic alterations between pre-treatment and post-treatment. However, the emergence of *de novo* alterations was observed in less than half of the patients with mCSPC and mCRPC after treatment. Recently, Herberts et al. performed deep whole-genome sequencing of serial plasma and synchronous metastases in patients with mCRPC, and found copy number gain of *AR* in addition to copy number loss of *TP53*, *RB1*, and *PTEN* after resistance to treatment targeting AR signaling, suggesting an important role of altered function of these genes in treatment resistance [32]. Consistently, this study observed *de novo* genomic alterations in *AR*, *TP53*, *RB1*, and *PTEN* in some patients after treatment. Schweizer et al. reported high concordance of DNA repair gene alterations in paired ctDNA and/or metastatic tissue when clonal hematopoiesis of indeterminate potential was excluded [13]. Consistently, *de novo* alterations in HRR genes were rare in this study, indicating that DNA repair gene alterations represent truncal events. In addition, this study showed that TF between pre-treatment and post-treatment was correlated in mCSPC, but not mCRPC, suggesting dynamic change of TF by treatment in mCRPC.

This study reported several interesting findings that add practical value beyond currently used clinical factors. This study showed the significance of genomic alterations as prognosticators in mCSPC and mCRPC. In mCSPC, high TF as well as *TP53* and *PTEN* alterations and NOTCH pathway alteration were prognostic factors for high risk of rapid progression to CRPC. Recently, it was reported that high ctDNA fraction was associated with poor OS in mCSPC, supporting the finding in this study [17, 33]. In addition, Stopsack et al. reported that genomic alterations in signaling of AR, cell cycle, MYC, TP53, and NOTCH by MSK-IMPACT panel using tissues were associated with shorter time to CRPC in mCSPC although detailed information on treatment is not available due to retrospective data-based analyses [21]. In addition, recent research using pre-treatment tumor tissues revealed that lower activity of NOTCH pathway was associated with primary resistance to ARPI in mCRPC, suggesting inactivation of NOTCH pathway play important role in obtaining treatment resistance in prostate cancer [34]. Consistently, our prospective observational study confirmed the adverse effect of *TP53* alteration and NOTCH pathway alteration and suggested the adverse effect of *PTEN* alteration by liquid biopsy on time to CRPC in mCSPC. In mCRPC, this study showed that *AR* alterations were associated with shorter treatment duration with ARPI, which is consistent with previous reports [14, 16, 17].

Notably, this study suggested that genomic alteration could be predictive of treatment efficacy in mCSPC. In mCSPC, HRR defect was associated with a different risk of progression to CRPC between ADT/CAB and ADT plus ARPI. This study showed that HRR defect was associated with a higher progression risk with ADT/CAB, but not with ADT plus ARPI in mCSPC, indicating that patients with HRR defect in mCSPC receive significant benefits from ARPI combination therapy.

This study evaluated genomic alterations in 324 genes using F1LCDx® and F1CDx®. However, a more comprehensive genome-wide determination needs to be conducted to capture somatic variants and tumor fractions more accurately. In addition, F1LCDx® and F1CDx® do not distinguish somatic and germline gene mutations, which limited an analysis and an interpretation of the findings. However, the result obtained from an investigation using commercially available cancer genomic testing showed usefulness in the clinical setting. Also, the number of prostate cancer patients enrolled in this study was not large, and the case number of some subgroups was small. This exploratory study did not adjust multiple testing, resulting in increased false discovery rate. Therapeutic intervention and clinical examination were not defined because the design was an observational study. The finding obtained in this study are exploratory, and need to be validated in the future.

This study revealed the genomic landscape of ctDNA in metastatic prostate cancer across the broad spectrum from mCSPC to mCRPC, accompanied by genomic profiles between tissue and ctDNA and clonal evolution during treatment. In addition, the impact of genomic signatures and alterations on prognosis and treatment efficacy was presented in mCSPC and mCRPC. These findings are valuable in the clinical practice of advanced prostate cancer. In particular, predictive factors such as HRR defect in mCSPC should be validated in the future.

## Supplementary information

Supplementary Figure 1

Supplementary Figure 2

Supplementary Table 1

Supplementary Table 2

Supplementary Table 3

Supplementary Table 4

## Data Availability

The datasets analyzed during the current study are available from the corresponding author on reasonable request.
