# Supplementary material for: Genomic profiling and clinical utility of circulating tumor DNA in metastatic prostate cancer: SCRUM-Japan MONSTAR SCREEN project
Source: BJC Rep. 2024 Apr 3;2:28. doi: 10.1038/s44276-024-00049-7 (PMC11523993; doi:10.1038/s44276-024-00049-7)
Supplement: Supplementary file 1 — Supplementary Figure 1 [file 44276_2024_49_MOESM1_ESM.pptx]

## Slide 1
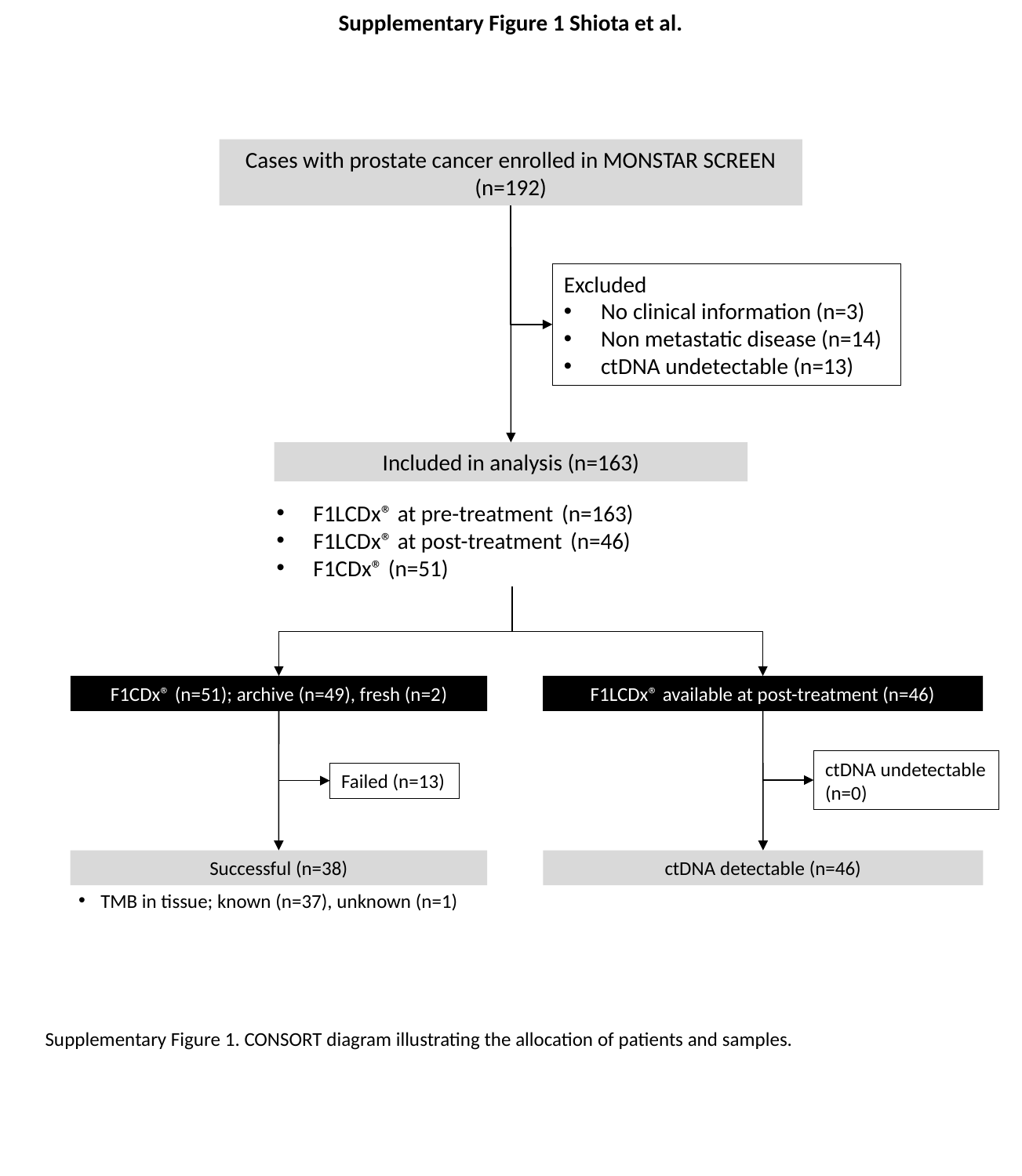

Supplementary Figure 1 Shiota et al.
Cases with prostate cancer enrolled in MONSTAR SCREEN
(n=192)
Excluded
No clinical information (n=3)
Non metastatic disease (n=14)
ctDNA undetectable (n=13)
Included in analysis (n=163)
F1LCDx® at pre-treatment (n=163)
F1LCDx® at post-treatment (n=46)
F1CDx® (n=51)
F1CDx® (n=51); archive (n=49), fresh (n=2)
Failed (n=13)
Successful (n=38)
TMB in tissue; known (n=37), unknown (n=1)
F1LCDx® available at post-treatment (n=46)
ctDNA undetectable (n=0)
ctDNA detectable (n=46)
Supplementary Figure 1. CONSORT diagram illustrating the allocation of patients and samples.
