# Supplementary material for: Genomic profiling and clinical utility of circulating tumor DNA in metastatic prostate cancer: SCRUM-Japan MONSTAR SCREEN project
Source: BJC Rep. 2024 Apr 3;2:28. doi: 10.1038/s44276-024-00049-7 (PMC11523993; doi:10.1038/s44276-024-00049-7)
Supplement: Supplementary file 2 — Supplementary Figure 2 [file 44276_2024_49_MOESM2_ESM.pptx]

## Slide 1
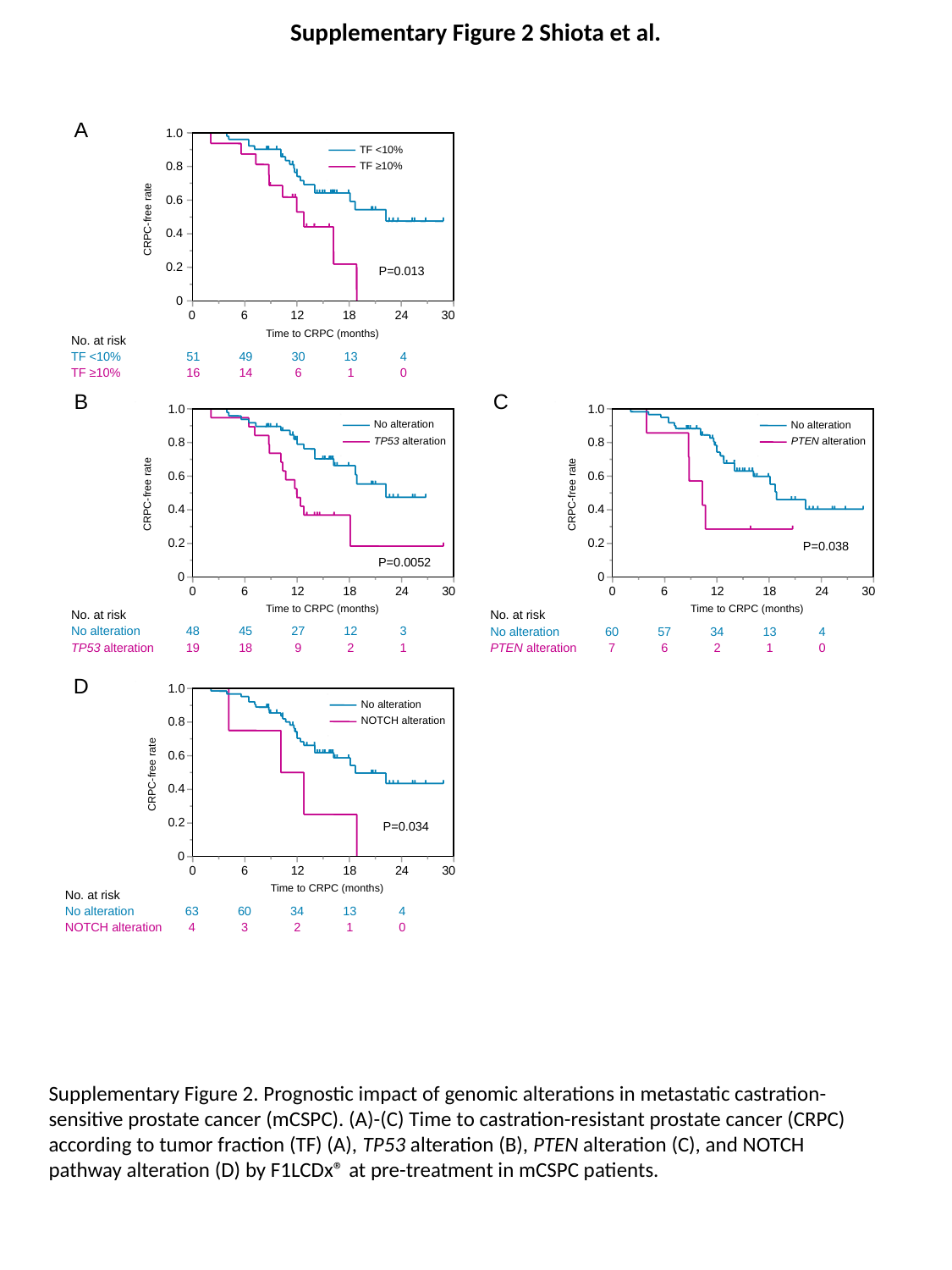

Supplementary Figure 2 Shiota et al.
A
1.0
TF <10%
TF ≥10%
0.8
0.6
CRPC-free rate
0.4
0.2
P=0.013
0
0
6
12
18
24
30
Time to CRPC (months)
| No. at risk | | | | | |
| --- | --- | --- | --- | --- | --- |
| TF <10% | 51 | 49 | 30 | 13 | 4 |
| TF ≥10% | 16 | 14 | 6 | 1 | 0 |
C
B
1.0
0.8
0.6
0.4
0.2
0
0
6
12
18
24
30
1.0
0.8
0.6
0.4
0.2
0
0
6
12
18
24
30
No alteration
TP53 alteration
No alteration
PTEN alteration
CRPC-free rate
CRPC-free rate
P=0.038
P=0.0052
Time to CRPC (months)
Time to CRPC (months)
| No. at risk | | | | | |
| --- | --- | --- | --- | --- | --- |
| No alteration | 48 | 45 | 27 | 12 | 3 |
| TP53 alteration | 19 | 18 | 9 | 2 | 1 |
| No. at risk | | | | | |
| --- | --- | --- | --- | --- | --- |
| No alteration | 60 | 57 | 34 | 13 | 4 |
| PTEN alteration | 7 | 6 | 2 | 1 | 0 |
D
1.0
0.8
0.6
0.4
0.2
0
0
6
12
18
24
30
No alteration
NOTCH alteration
CRPC-free rate
P=0.034
Time to CRPC (months)
| No. at risk | | | | | |
| --- | --- | --- | --- | --- | --- |
| No alteration | 63 | 60 | 34 | 13 | 4 |
| NOTCH alteration | 4 | 3 | 2 | 1 | 0 |
Supplementary Figure 2. Prognostic impact of genomic alterations in metastatic castration-sensitive prostate cancer (mCSPC). (A)-(C) Time to castration-resistant prostate cancer (CRPC) according to tumor fraction (TF) (A), TP53 alteration (B), PTEN alteration (C), and NOTCH pathway alteration (D) by F1LCDx® at pre-treatment in mCSPC patients.
